# Pathological RANK signaling in B cells drives autoimmunity and chronic lymphocytic leukemia

**DOI:** 10.1084/jem.20200517

**Published:** 2020-10-14

**Authors:** Begüm Alankus, Veronika Ecker, Nathalie Vahl, Martina Braun, Wilko Weichert, Stephan Macher-Göppinger, Torben Gehring, Tanja Neumayer, Thorsten Zenz, Maike Buchner, Jürgen Ruland

**Affiliations:** 1 Institut für Klinische Chemie und Pathobiochemie, Klinikum rechts der Isar, Technical University of Munich, Munich, Germany; 2 TranslaTUM, Center for Translational Cancer Research, Technical University of Munich, Munich, Germany; 3 Institute of Pathology, Technical University of Munich, Munich, Germany; 4 German Cancer Consortium, Heidelberg, Germany; 5 Institute of Pathology, University Medical Center Mainz, Mainz, Germany; 6 Department of Medical Oncology and Hematology, University Hospital and University of Zurich, Zurich, Switzerland; 7 German Center for Infection Research, Munich, Germany

## Abstract

Clinical evidence suggests alterations in receptor activator of NF-κB (RANK) signaling are key contributors to B cell autoimmunity and malignancy, but the pathophysiological consequences of aberrant B cell–intrinsic RANK signaling remain unknown. We generated mice that express a human lymphoma–derived, hyperactive RANK^K240E^ variant in B lymphocytes in vivo. Forced RANK signaling disrupted B cell tolerance and induced a fully penetrant systemic lupus erythematosus–like disease in addition to the development of chronic lymphocytic leukemia (CLL). Importantly, RANK^K240E^ transgenic CLL cells as well as CLL cells of independent murine and of human origin depend on microenvironmental RANK ligand (RANKL) for tumor cell survival. Consequently, inhibition of the RANKL–RANK axis with anti-RANKL antibodies killed murine and human CLL cells in vitro and in vivo. These results establish pathological B cell–intrinsic RANK signaling as a potential driver of autoimmunity and B cell malignancy, and they suggest the exploitation of clinically available anti-RANKL compounds for CLL treatment.

## Teaser

Enforced RANK signaling in B cells drives systemic autoimmunity and CLL development. The survival of CLL cells depends on microenvironmental RANKL, and inhibition of RANKL–RANK signaling kills murine and human CLL cells.

## Introduction

B lymphocytes are critical for adaptive immunity and host protection against infection (LeBien and Tedder, 2008), but when dysregulated they can also drive autoimmunity or develop into malignant lymphomas (Goodnow, 2007; Kwak et al., 2019; Nemazee, 2017; Nogai et al., 2011; Taher et al., 2017). The normal development of B cells in the bone marrow and their activation and expansion in the periphery are controlled by signals from the B cell antigen receptor (BCR; Kurosaki et al., 2010; Taher et al., 2017). Additional signals from dedicated coreceptors are required to mobilize productive immunity, since B cell engagement by antigen alone has only a limited capacity to activate the crucial PI3K/AKT and NF-κB pathways for lymphocyte growth and survival. Instead, BCR engagement alone induces inhibitory feedback mechanisms that result in B cell anergy, which is one mechanism that prevents autoreactive B cell activation after self-antigen sensing. Additional tolerance checkpoints during B cell differentiation further prevent self-reactive B cell activity by restricting BCR signaling to the prosurvival factors PI3K/AKT, NF-κB, and BCL-2 in immature cells (reviewed in Goodnow, 2007). Pathological mechanisms that disrupt or overwrite these tolerance checkpoints can result in severely debilitating autoimmune diseases such as systemic lupus erythematosus (SLE), rheumatoid arthritis (RA), and Sjogren’s syndrome (reviewed in Goodnow, 2007). Additionally, large epidemiological studies have demonstrated an increased incidence of B cell malignancies in patients with such autoimmune conditions (Bernatsky et al., 2006). While these data suggest that the molecular pathways that drive B cell autoimmunity and B cell lymphoma overlap, the underlying molecular mechanisms are still insufficiently defined.

Members of the TNF receptor superfamily (TNFRSF) constitute a family of B cell coreceptors that synergize with the BCR to enhance clonal lymphocyte proliferation and survival for the host defenses (Rickert et al., 2011). Prominent examples are CD40 and B cell–activating receptor. Loss-of-function mutations in these molecules are causally connected to immunodeficiencies in mouse models and humans, and gain-of-function alterations are associated with autoimmunity and B cell malignancy (Batten et al., 2004; Rickert et al., 2011; Smulski and Eibel, 2018). An additional TNFRSF member with emerging potential roles in B cell immunopathology is the receptor activator of NF-κB (RANK; also designated TNFRSF11A). RANK expression can be induced on B lymphocytes (Anderson et al., 1997; Yun et al., 1998) but is also expressed on other cell types, such as osteoclast precursors and mature osteoclasts or mammary epithelial cells (Walsh and Choi, 2014). RANK activation by cell-bound or soluble forms of RANK ligand (RANKL, also designated TNFSF11) induces receptor trimerization and, similar to other TNFRSF members, recruits TNF receptor–associated factors with activation of PI3K and MAP kinases as well as canonical and noncanonical NF-κB signaling (Kim et al., 2009; Wada et al., 2006; Walsh and Choi, 2014).

Systemically increased active levels of RANKL are detected in the sera of SLE patients, and increased local concentrations are found in the synovial joint fluids of RA patients, both of which are positively correlated with disease severity (Carmona-Fernandes et al., 2011; Fonseca et al., 2005). In addition, single-nucleotide polymorphisms in either the *TNFRSF11A* locus (encoding RANK) or the *TNFSF11* locus (encoding RANKL) are associated with the autoimmune syndromes myasthenia gravis and autoimmune vitiligo, respectively (Jin et al., 2016; Renton et al., 2015). Furthermore, the malignant B cells in Hodgkin’s lymphoma frequently coexpress RANK and RANKL, which are thought to mediate autocrine or paracrine survival signaling, and chronic lymphocytic leukemia (CLL) B cells have been reported to express RANK at increased levels compared with normal B cells (Schmiedel et al., 2013; Secchiero et al., 2006; Wierda et al., 2003). A possible role of RANK in bone remodeling was suggested in CLL (Marini et al., 2017). Finally, somatically acquired mutations in the intracellular signaling domain of RANK (change of Lys to Glu at amino acid position 240; K240E) are recurrently detected in human diffuse large B cell lymphoma specimens (Compagno et al., 2009; Davis et al., 2010; Wilson et al., 2015), and these mutations have been suggested to lead to gain-of-function modifications (Davis et al., 2010). While all these correlative clinical data together indicate that alterations in the RANKL–RANK signaling axis may contribute to B cell autoimmunity and malignancy, the pathophysiological consequences of deregulated RANK signaling in B cells remain unknown.

To study forced RANK signaling in B cells in vivo, we conditionally expressed a human lymphoma–derived RANK^K240E^ variant in mice. Surprisingly, we found that B cell–intrinsic RANK^K240E^ signaling was sufficient to drive a fully penetrant SLE-like autoimmune disease and facilitated B cell transformation and CLL development, and the tumor cells depended on RANKL from the microenvironment. The RANKL–RANK axis also promoted tumor viability in human CLL models and primary patient samples, which could be disrupted with clinically available anti-RANKL antibodies.

## Results

### RANK^K240E^ expression induces ligand-dependent B cell activation with B1 cell expansion

**Figure 1.**
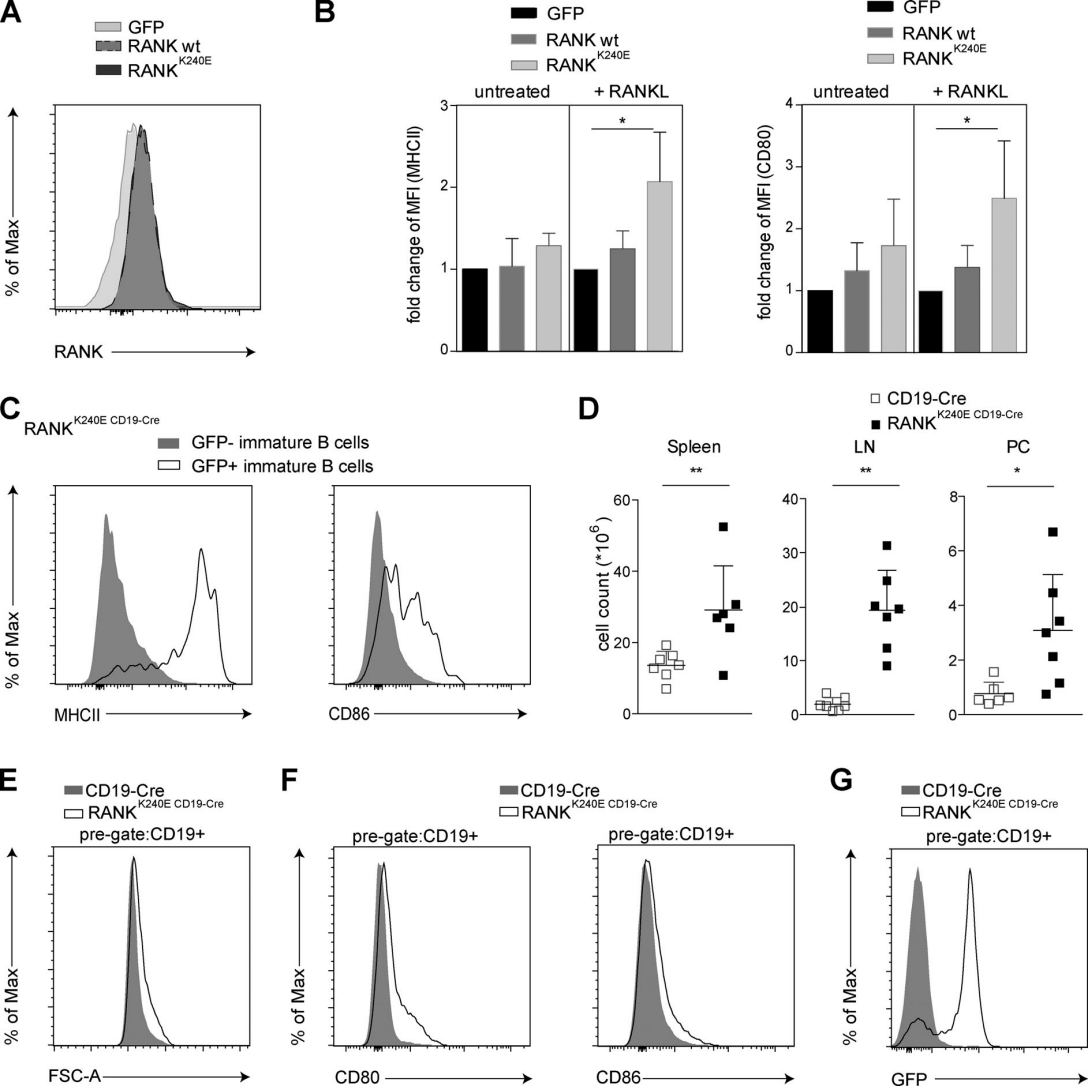
**Effects of RANK^K240E^ expression on B cells in vitro and in vivo. **Bal17 cells were infected with retroviruses to express empty vector (GFP), RANK wt, and RANK^K240E^ and sorted for GFP^+^ cells. **(A)** The surface RANK expression in Bal17 cells transduced with wt RANK or RANK^K240E^ was equal as determined by flow cytometry; representative of two independent experiments. **(B)** The empty vector (GFP), RANK wt, and RANK^K240E^-expressing Bal17 cells were subsequently stimulated with 100 ng/ml RANKL for 1 h. Activated surface marker expression (CD80 and MHCII) was measured using flow cytometry. The pooled analysis from three independent experiments is shown with a relative increase in the mean fluorescence intensity (MFI) compared with the empty vector control (GFP). For statistical analysis, Student’s *t* test was performed. *, P < 0.05. **(C)** Immature B cell activation upon RANK^K240E^ expression. Cells were pregated on immature B cells (B220^+^AA4.1^+^), and RANK^K240E^/GFP-expressing cells were distinguished by the expression of GFP. The data shown are representative of five independent experiments, with a total number of at least 12 mice analyzed per genotype. **(D)** Total B cell counts in the spleen, LN, and peritoneal cavity (PC) as determined by CD19^+^ lymphocytes; the data shown are representative of three independent experiments, with a total number of at least six mice analyzed per genotype; dot plot graphs are shown for individual mice, and bars indicate the means with SD; Student’s *t* test was performed. *, P < 0.05; **, P < 0.01. **(E and F)** RANK^K240E^ expression mediates an activated B cell phenotype in vivo: CD80, CD86, and forward-scatter area (FSC-A) expression were measured using flow cytometry of splenic B cells. The data shown are representative of four independent experiments, with a total number of at least eight mice analyzed per genotype. **(G)** RANK^K240E^ expression is indicated by GFP expression; representative example for at least *n* = 30 mice analyzed.

**Figure S1.**
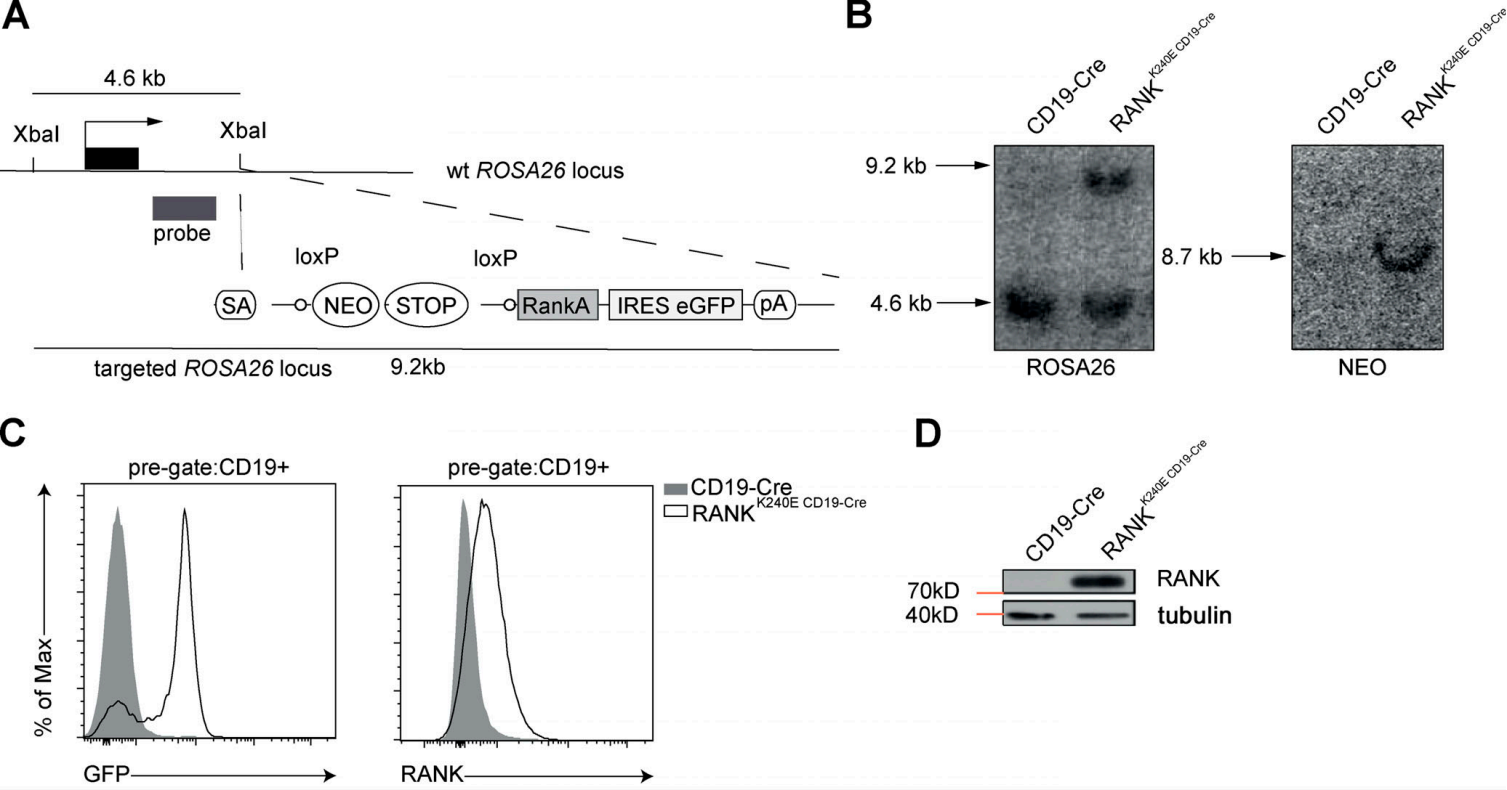
**Generation of RANK^K240E^**-**expressing mice.** **(A)** RANK^K240E^ targeting strategy. NEO, neomycin; pA, poly(A); SA, splice acceptor;** (B)** Southern blot analysis is shown. Lanes 1 and 2 show genomic DNA from wt and transgenic mice. The size of the fragment showing the wt ROSA26 locus is 4.6 kb, the recombined locus is 9.2 kb, and Cre-mediated removal of the STOP cassette results in an 8.7-kb fragment. **(C)** GFP and surface RANK expression in B cells in the spleens of 2-mo-old RANK^K240E CD19-Cre^ and wt mice, as analyzed by flow cytometry. The cells were pregated on CD19. Representative of three mice analyzed in three independent experiments. **(D)** Western blot analysis of MACS-purified B cells shows overexpression of RANK^K240E^ in transgenic B cells, representative for two independent experiments.

To study pathological RANK signaling in B cells in vivo, we first explored whether the human lymphoma–derived RANK^K240E^ variant (Davis et al., 2010) could be used as a tool in murine cells. To this end, we transduced RANK^K240E^ and wild-type (wt) RANK into the murine Bal17 B cell line (Fig. 1 A). Both wt RANK and RANK^K240E^ were expressed at the same level and were not sufficient by themselves to induce B cell activation, as determined by the expression of the B cell activation markers CD80 and MHCII. Upon exogenous RANKL stimulation, both RANK variants triggered B cell activation, but RANK^K240E^-expressing Bal17 cells exhibited a substantially stronger increase in CD80 and MHCII expression than wt RANK-expressing cells (Fig. 1 B), indicating hyperactivity of the RANK^K240E^ version. Encouraged by these results, we next created a transgenic mouse line for inducible RANK^K240E^ expression by introducing the human RANK^K240E^ cDNA preceded by a loxP-flanked transcriptional and translational STOP cassette into the ubiquitously expressed Rosa26 locus (Pechloff et al., 2010). The resulting Rosa26^loxSTOPlox^RANK^K240E^ mice were crossed with CD19-Cre transgenic animals for B cell–specific excision of the STOP cassette (Rickert et al., 1997), leading to B cell–restricted RANK^K240E^ expression. Enhanced GFP is coexpressed with RANK^K240E^ from an internal ribosomal entry site (IRES) in double transgenic offspring (referred to as RANK^K240E CD19-Cre^ mice), which enables monitoring of the RANK^K240E^-expressing cells (Fig. S1, A–D).

**Figure S2.**
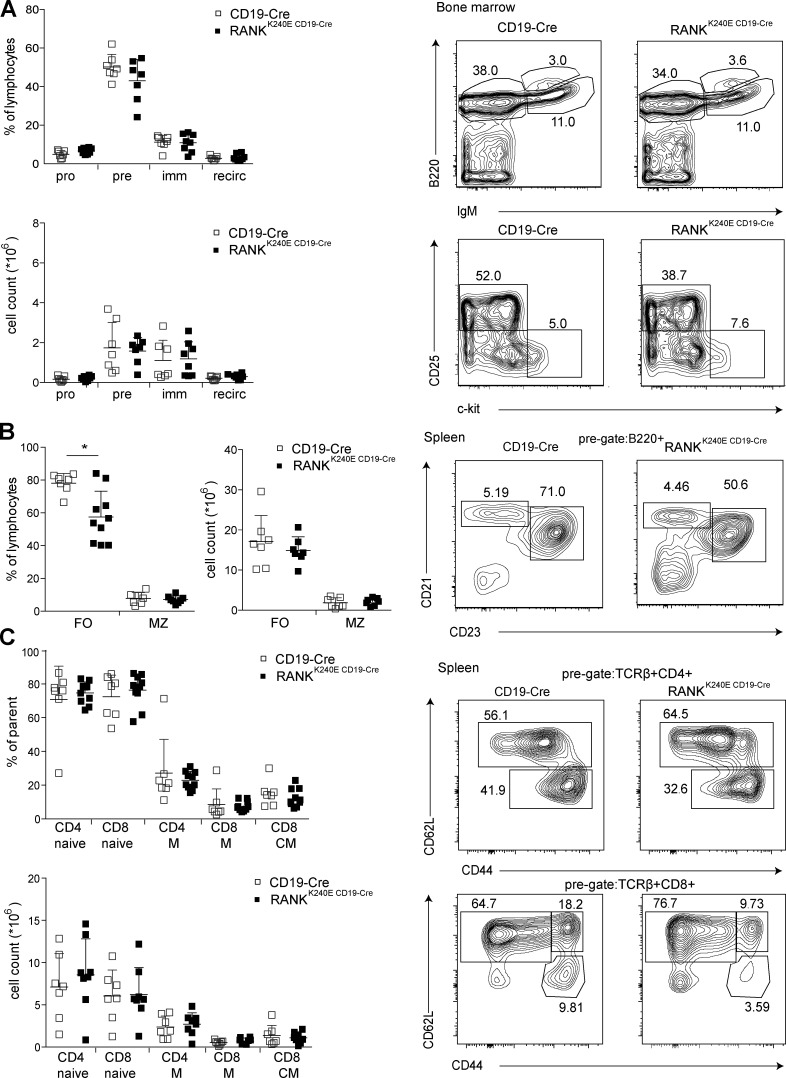
**Analysis of 2–6-mo-old RANK^K240E CD19-Cre^ and control mice.** **(A)** The bone marrow of RANK^K240E CD19-Cre^ (*n* = 7) and control littermates (*n* = 7) was analyzed for B cell developmental stages in at least three independent experiments. Pro– (B220^+^ IgM^−^ cKit^+^ CD25^−^), pre– (B220^+^ IgM^−^ cKit^−^ CD25^+^), immature (imm; B220^+^ IgM^+^), and recirculating (recirc; B220^hi^ IgM^+^) B cells are summarized, and the quantification of relative and absolute values is shown (left), as is a representative FACS plot (right). **(B)** The splenocytes of RANK^K240E CD19-Cre^ (*n* ≥ 7) and control littermates (*n* ≥ 7) were analyzed for marginal zone (MZ) and follicular (FO) B cell content in at least three independent experiments. The quantification of relative and absolute values is shown, as is a representative FACS plot. **(C)** Splenocytes were analyzed for T cell subsets of RANK^K240E CD19-Cre^ (*n* ≥ 7) and control littermates (*n* ≥ 7), analyzed in at least three independent experiments as depicted in the representative FACS plot (right) and quantified in the dot plot graph (left). For statistical analysis, Student’s *t* test was performed. *, P < 0.05.

In the bone marrow of 6–12-wk-old RANK^K240E CD19Cre^ mice, we detected a regular composition of early pro–, pre–, immature, and recirculating B cell populations (Fig. S2 A). The percentage of B220^+^AA4.1^+^ immature B cells was comparable to that of littermate control mice, but the RANK^K240E^-expressing B cells showed aberrant surface expression of MHCII and CD86, indicating active RANK^K240E^ signaling (Fig. 1 C). In the periphery, we observed elevated numbers of RANK^K240E^-expressing B cells in the spleens (∼2-fold), lymph nodes (∼10-fold), and peritoneal cavities (∼8-fold; Fig. 1 D), with an activated phenotype characterized by a larger cell size and elevated CD80 and CD86 surface expression and confirmed GFP expression (Fig. 1, E–G).

**Figure 2.**
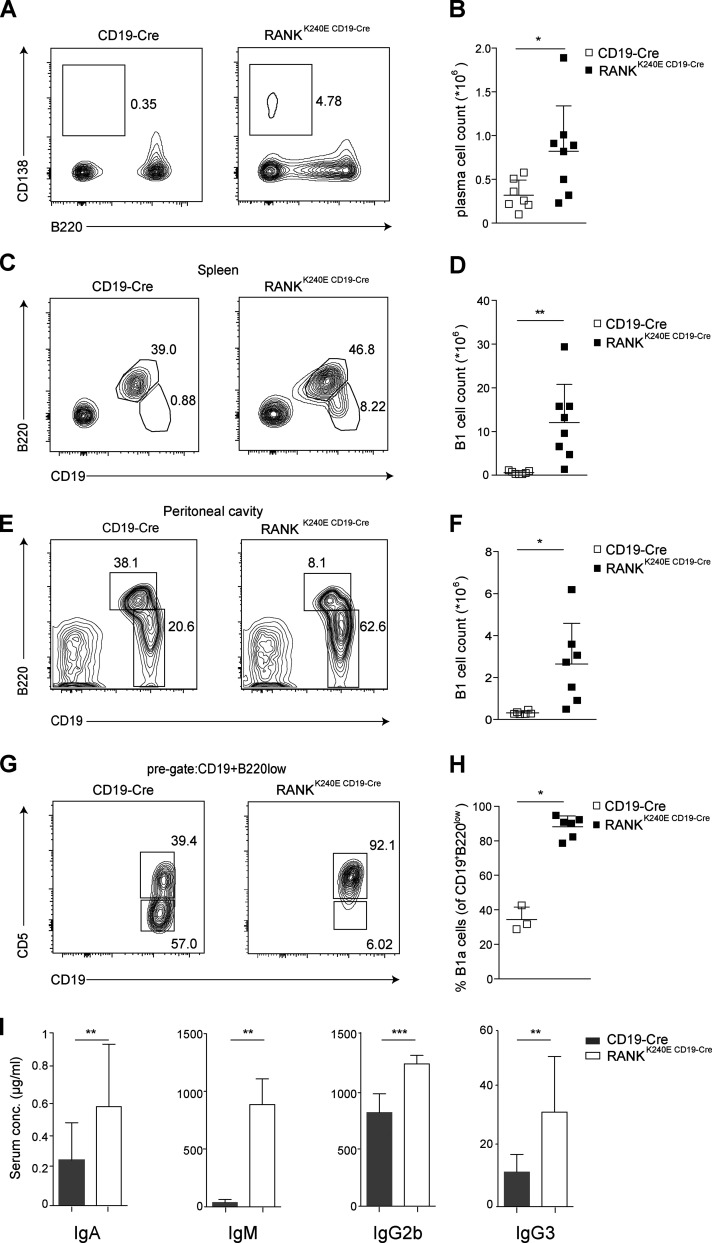
**RANK^K240E^ expression mediates B cell differentiation and antibody production.** **(A)** The increased percentage of plasma cells in the spleen of 6-mo-old RANK^K240E CD19-Cre^ mice is shown with a representative FACS plot. **(B)** Summary of the total cell counts of plasma cells in the spleens of 8–12-wk-old RANK^K240E CD19-Cre^ mice (*n* = 8) and littermate controls (*n* = 7) are shown as a dot plot graph with mean + SD indicated. Analysis was performed in five independent experiments. **(C)** Representative analysis of B1 and B2 cell populations in the spleen by flow cytometry (6-mo-old animal). **(D)** Dot plot graph depicting the absolute B1 cell count in the spleen of 6–12-wk-old RANK^K240E CD19-Cre^ (*n* = 8) and control (*n* = 7) mice. Analysis was performed in five independent experiments. **(E)** Representative analysis of B1 and B2 cell populations in the peritoneum of 6-mo-old RANK^K240E CD19-Cre^ mice and littermate control by flow cytometry. **(F)** Dot plot graph depicting the absolute B1 cell count in the peritoneum of 6–12-wk-old RANK^K240E CD19-Cre^ (*n* = 8) and control (*n* = 7) mice. Analysis was performed in five independent experiments. **(G)** B1a cell expansion in RANK^K240E CD19-Cre^ mice revealed by flow cytometry. The cells were pregated for the CD19^+^B220^low^ B1 population, and a representative FACS plot for CD5 expression is shown. **(H)** Dot plot graph is depicting the relative B1a cell count in the peritoneum of 6–12-wk-old RANK^K240E CD19-Cre^ (*n* = 6) and control (*n* = 3) mice. Analysis was performed in two independent experiments. **(I) **Basal serum levels of IgA, IgM, IgG2b, and IgG3 in 3-mo-old mice (*n *≥ 5 per genotype); analysis was performed in two independent experiments. The data are shown as the mean with SEM. For statistical analysis, Student’s *t* test was performed. *, P < 0.05; **, P < 0.01.

While the numbers of follicular and marginal zone B cells were not altered in RANK^K240E^ mice (Fig. S2 B), the B220^low^CD138^hi^ plasma cell population was significantly increased (Fig. 2, A and B), as was the frequency of splenic and peritoneal CD19^+^B220^low^ B1 cells (Fig. 2, C–F). These B1 cells were predominately of the CD5^+^ B1a subtype (Fig. 2, G and H). The frequencies and numbers of naive, memory, and central memory CD4^+^ and CD8^+^ T cells were not altered (Fig. S2 C). Thus, experimentally enforced B cell intrinsic RANK^K240E^ expression drives premature B cell activation with an accumulation of plasma cells and B1a cells in vivo. In line with these findings, we also observed an increase in the total concentrations of IgA, IgM, IgG2b, and IgG3 in the sera of 3-mo-old RANK^K240E CD19-Cre^ mice (Fig. 2 I).

### RANK^K240E^ expression in B cells drives lymphoproliferative autoimmune disease

**Figure 3.**
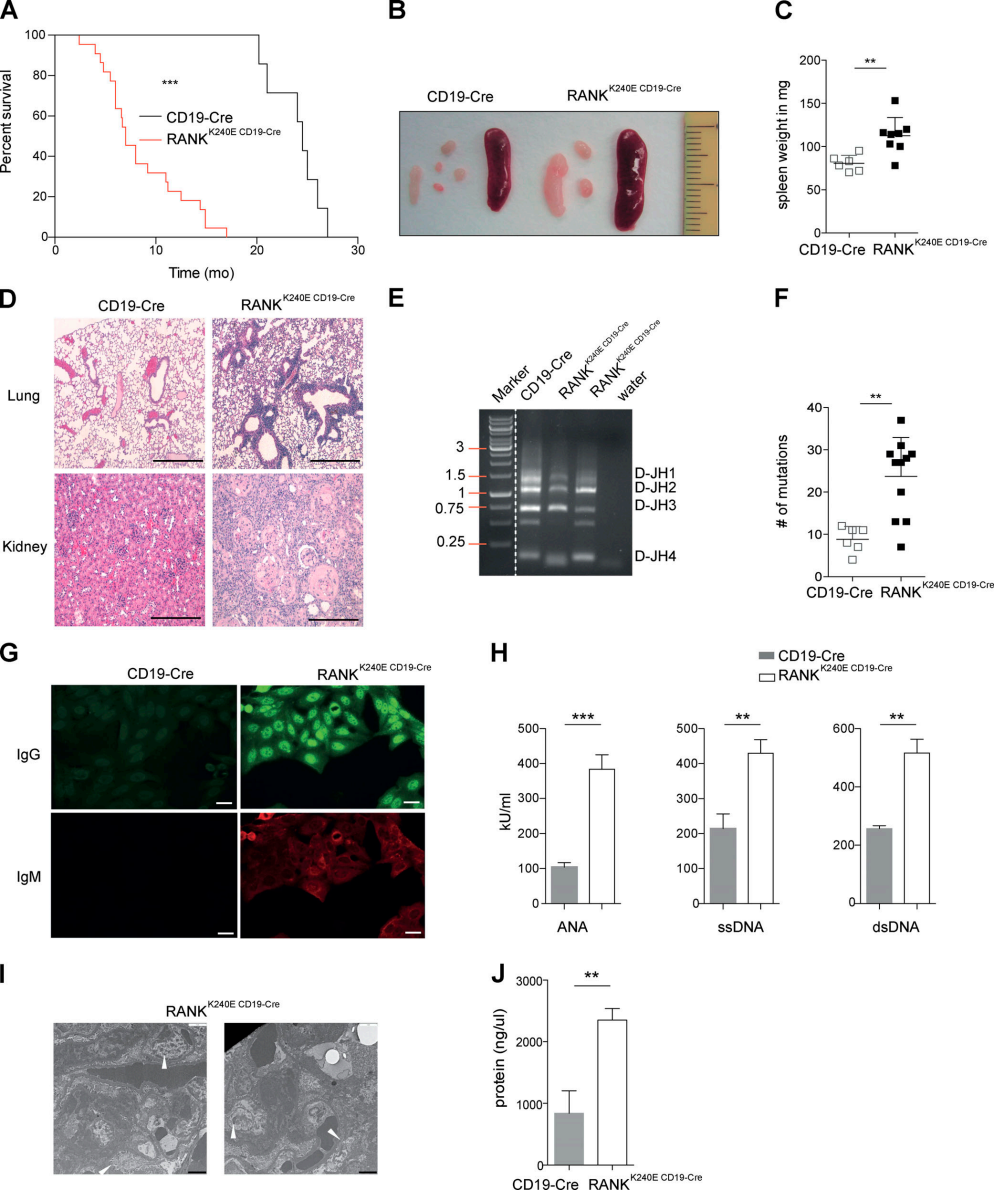
**RANK^K240E^ expression drives disease in vivo. (A)** Kaplan–Meier curve of CD19-Cre and RANK^K240E CD19-Cre^ mice (*n* = 22 for RANK^K240E CD19-Cre^ mice and *n* = 7 for CD19-Cre mice). For statistical analysis, log-rank (Mantel Cox) analysis was performed. ****, P < 0.0001. **(B)** Macroscopic appearance of representative spleens and mesenteric lymph nodes (in centimeters), representative example for RANK^K240E CD19-Cre^ and littermate control mice analyzed for* n *≥ 5 per genotype. **(C)** Dot plot graph depicts spleen weight for RANK^K240E CD19-Cre^ (*n* = 8) and littermate control (*n* = 5) mice is shown (<6 mo of age). **(D)** Representative histological analysis of* n *≥ 3 per genotype mice analyzed revealed glomerulonephritis-related kidney destruction and cellular infiltration of lungs in RANK^K240E CD19-Cre^ mice were revealed by H&E staining (scale bars, 1 mm). **(E)** Ig clonality analysis by PCR of genomic DNA isolated from GFP^+^-sorted B cells, representative of seven mice (6 mo of age) per genotype. **(F)** SHM analysis of 4-mo-old mice by PCR of genomic DNA isolated from GFP^+^-sorted B cells derived from four mice per genotype, revealing a significantly increased mutation frequency in RANK^K240E CD19-Cre^ mice. Fragments corresponding to the V_H_J558-J_H_4 region were amplified and sequenced, and the mutation rate of the V region (∼300 bp) was determined by IMGT analysis. **(G)** Detection of high levels of antinuclear IgG in RANK^K240E CD19-Cre^ mice. Autoreactive antibodies in sera of CD19-Cre and RANK^K240E CD19-Cre^ mice (>3 mo of age) using HEp-2 cells were detected by indirect immunofluorescence. Secondary detection antibodies were labeled with FITC (anti-IgG) or APC (anti-IgM). Representative example is shown for *n* = 4 per genotype, analyzed in two independent experiments. Scale bars, 25 µm. **(H)** Basal serum levels of total ANAs, anti-ssDNA (single-strand DNA) antibodies, and anti-dsDNA (double-strand DNA) antibodies in 6-mo-old mice (*n *≥ 4 per genotype), analyzed using Alpha Diagnostic International Autoimmune ELISA kits in two independent experiments. **(I)** Scanning electron microscopy images of kidneys in terminally ill 6-mo-old RANK^K240E CD19-Cre^ mice showing massive electron-dense (immune) deposit accumulation (indicated by arrowheads, representative of three mice; scale bars, 3 µm). **(J)** Kidney destruction in RANK^K240E CD19-Cre^ mice is also evident in the increased proteinuria (*n* = 6 per genotype). The data shown were obtained from terminally ill 6-mo-old animals. For statistical analysis, Student’s *t* test was performed. **, P < 0.01; ***, P < 0.001.

Next, we followed a cohort of RANK^K240E CD19-Cre^ mice over time. Intriguingly, all animals developed fatal lymphoproliferative disease (Fig. 3 A) with splenomegaly, lymphadenopathy (Fig. 3, B and C), and pathological lymphocyte infiltration into organs such as the lungs and kidneys, leading to a disruption of normal tissue architecture (Fig. 3 D). The expanded B cells were of polyclonal origin (Fig. 3 E) and showed a two- to threefold increase in the mutation frequency of their variable heavy (V_H_) region compared with littermate control B cells, indicating that they had undergone somatic hypermutations (SHMs; Fig. 3 F). Furthermore, indirect immunofluorescence assays using HEp-2 cells indicated the presence of autoreactive antibodies in the sera of RANK^K240E CD19-Cre^ mice (Fig. 3 G). Similarly, we detected high concentrations of autoimmune antinuclear antibodies (ANAs) against single- and double-stranded DNA by ELISA (Fig. 3 H). Finally, we found mesangial and subendothelial immune-complex depositions in the kidneys (Fig. 3 I) and massive proteinuria as a sign of kidney damage in RANK^K240E CD19-Cre^ mice but not in control littermate mice (Fig. 3 J). Thus, pathological RANK^K240E^ signaling in B cells disrupts immune tolerance and promotes the expansion and activation of B cell clones with high-affinity autoimmune BCRs (Detanico et al., 2013; Guo et al., 2010), resulting in a systemic autoimmune disorder with key hallmarks of human SLE, such as ANAs and terminal kidney damage (Tsokos, 2011).

### RANK^K240E^-expressing B cells use RANKL from the microenvironment for survival and proliferation

**Figure 4.**
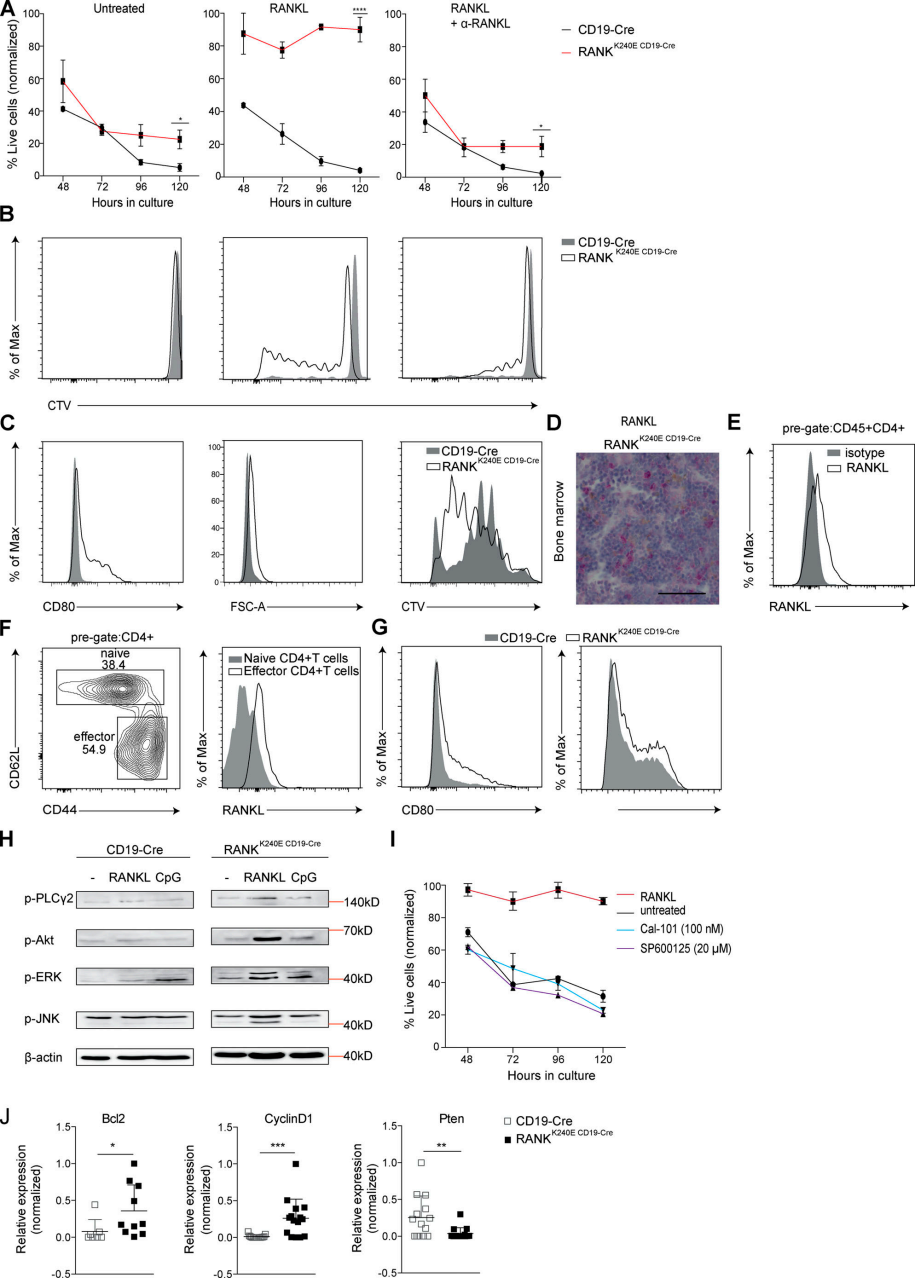
**RANK^K240E^**-**expressing B cells are dependent on RANKL for survival and proliferation in vitro.** **(A and B) **GFP^+^-sorted B cells from 2–4-mo-old RANK^K240E CD19-Cre^ and CD19-Cre control mice were stained with CellTrace Violet proliferation dye and kept in culture in the presence or absence of RANKL or α-RANKL for 5 d. Flow cytometry analysis of Annexin V– and 7-AAD–stained B cells revealed enhanced survival and proliferation in the presence of RANKL. The viability was normalized to d0 (defined as 100%) for each individual mouse. The data shown are representative of four independent experiments with a total of eight mice per genotype. **(C)** GFP^+^-sorted B cells from 2–4-mo-old RANK^K240E CD19-Cre^ and CD19-Cre control mice were stained with CellTrace Violet proliferation dye, incubated with ST-2 bone marrow stromal cells for 72 h in culture, and analyzed for CD80 as a B cell activation marker, cell size, and cell proliferation by flow cytometry. The data shown are representative of three independent experiments with at least six mice per genotype. **(D)** Immunohistochemistry staining of bone marrow from 4-mo-old RANK^K240E CD19-Cre^ mice showing RANKL^+^ bone marrow stromal cells in RANK^K240E CD19-Cre^ mice. **(E)** RANKL surface expression was detected on CD45+CD4+ T cells in RANK^K240E CD19-Cre^ mice as compared with isotype control (anti-mouse IgG2a) staining. Representative analysis for a total of *n* = 5 mice is shown (summary for spleen and bone marrow depicted in Fig. S3, A and B). **(F)** MACS-isolated, CD3/CD28-activated CD4^+^ T cells were analyzed for RANKL surface expression by flow cytometry, and a representative example of three independent experiments is shown at 24 h after stimulation. **(G)** GFP^+^-sorted B cells from 2–4-mo-old RANK^K240E CD19-Cre^ and CD19-Cre control mice were incubated with magnetic bead-isolated, CD3/CD28-activated CD4^+^ T cells for 48 h and analyzed for CD80 and MHCII activation markers by flow cytometry (representative for *n* = 3 per genotype). **(H)** Immunoblots of GFP^+^-sorted B cells from the spleens of 4-mo-old mice. The data shown are representative of at least two independent experiments. **(I)** Pharmacological inhibition of the JNK pathway with SP600125 and the PI3K pathway with Cal-101 24 h after stimulation with RANKL led to drastically reduced cell survival in RANK^K240E^-expressing B cells (*n* ≥ 4 mice analyzed in two independent experiments). **(J)** Transcriptional expression levels of *Bcl-2*, *Cyclin-D1*, and *Pten* in sorted B cells of 2–4-mo-old RANK^K240E CD19-Cre^ and CD19-Cre littermate control mice (*n* ≥ 7 per genotype, determined in three independent experiments). For statistical analysis, Student’s *t* test was performed. *, P < 0.05; **, P < 0.01; ***, P < 0.001.

To understand the cellular effects of pathological RANK^K240E^ signaling in B cells, we next isolated B lymphocytes from RANK^K240E CD19-Cre^ and littermate control mice and cultured them in vitro*.* Even without exogenous stimulation, the RANK^K240E^-expressing B lymphocytes survived significantly better than wt B cells (Fig. 4 A, left), as determined by flow cytometric analysis. Over five days, B cells of both genotypes died progressively. However, upon exposure to exogenous RANKL, the RANK^K240E^-expressing B cells remained viable for at least 5 d (Fig. 4 A, center), and they proliferated vigorously in contrast to wt B cells even without additional mitogens or BCR costimulation (Fig. 4 B). These growth-promoting effects of RANKL were neutralized by α-RANKL antibodies (Fig. 4, A and B), demonstrating that the RANK^K240E^ signals are massively enhanced by exogenous RANKL stimulation.

**Figure S3.**
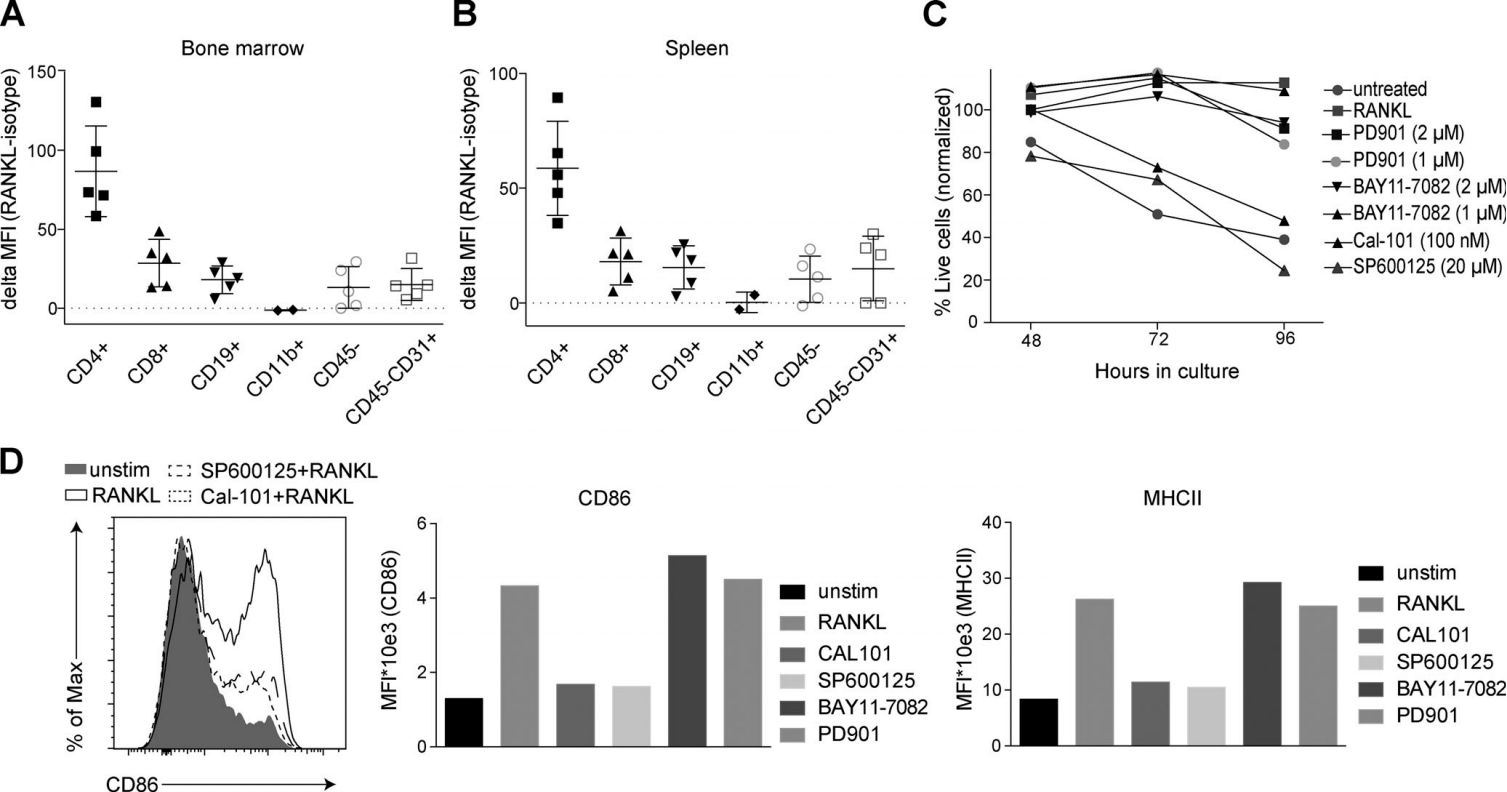
**RANKL expression and RANK^K240E^ survival pathway analysis. (A and B)** Flow cytometric analysis of bone marrow (A) and spleen (B) revealed high expression levels of RANKL on CD4^+^ T cells and no/low expression on CD8^+^ T cells (*n* = 5), CD19^+^ B cells (*n* = 5), CD11b^+^ myeloid cells (*n* = 2), CD45^−^ nonhematopoietic cells (*n* = 5), and CD45^−^CD31^+^ endothelial cells (*n* = 5), analyzed in two independent experiments. **(C)** Pharmacological inhibition of the MAPK pathway with PD901 and the NF-κB pathway with BAY11-7082 24 h after stimulation with RANKL led to minor changes in cell survival in RANK^K240E^-expressing B cells. Inhibition of the JNK pathway with SP600125 and the PI3K pathway with Cal-101 was included as a positive control (representative for *n* = 3 mice, analyzed in two independent experiments). **(D)** GFP+ B cells from RANK^K240E^ transgenic mice were FACS-sorted and treated with the indicated inhibitor for 30 min before adding recombinant RANKL. After 24 h, surface marker expression was analyzed. A representative FACS blot for *n* = 3 mice is shown, analyzed in two independent experiments (left). In addition, the MFI values of CD86 expression and MHCII expression upon treatment with RANKL with and without inhibitor cotreatment is shown, representative for *n* = 3, analyzed in two independent experiments.

Multiple cell types can provide RANKL to developing and mature B cells in vivo (Walsh and Choi, 2014), most prominently stromal cells in the bone marrow microenvironment and activated CD4^+^ T cells in peripheral tissues (Wang et al., 2002). To determine whether these cell types could stimulate RANK^K240E^ transgenic B cells, we first cocultured RANK^K240E^-expressing B lymphocytes with stroma-derived ST-2 cells that endogenously express RANKL (Nishida et al., 2005). In the presence of ST-2 cells, RANK^K240E^-expressing B cells, though not wt B cells from littermate control mice, acquired an activated phenotype, as indicated by CD80 expression. Moreover, their cell size increased, and RANK^K240E^-expressing B cells proliferated rapidly (Fig. 4 C). Immunohistochemistry analysis of bone marrow from RANK^K240E CD19-Cre^ mice further demonstrated that the bone marrow microenvironmental cells also strongly expressed RANKL in vivo in the direct vicinity of RANK^K240E^-expressing B cells (Fig. 4 D). Subsequent flow cytometric analysis revealed that primarily ex vivo isolated CD4^+^ T cells express RANKL in RANK^K240E CD19-Cre^ mice (Fig. 4 E and Fig. S3, A and B). In line with this finding, ex vivo–activated peripheral CD4^+^ T cells, which, as expected (Wang et al., 2002), up-regulated RANKL on the surface (Fig. 4 F), were also able to induce robust and selective activation of RANK^K240E^-expressing B cells but not wt B cells from littermate control mice (Fig. 4 G).

To gain mechanistic insights into the intracellular pathways that mediate the RANK^K240E^-induced pathological effects, we then stimulated RANK^K240E^ transgenic B cells with RANKL or the TLR9 agonist CpG as a control in vitro and performed Western blot analysis. RANKL stimulation induced the activation of the JNK and ERK pathways in RANK^K240E^-expressing cells but not in wt B cells from littermate control mice (Fig. 4 H). The PI3K/AKT pathway was also robustly activated (Fig. 4 H). Overall, JNK, ERK, and AKT activation by RANKL was more pronounced than that seen upon CpG stimulation in RANK^K240E^-expressing B cells, which is consistent with the notion that RANK^K240E^ is a strong signaling receptor (Fig. 4 H). Pharmacological inhibition of PI3K or JNK signaling, though not MEK or NF-κB inhibition, prevented the prosurvival and mitogenic effects of RANKL (Fig. 4 I and Fig. S3 C) and the activated B cell phenotype induced by RANK^K240E^ ligation (Fig. S3 D). These results together indicate that the activation of the PI3K and JNK pathways is particularly critical for the B cell–intrinsic effects of forced RANK^K240E^ signaling. To gain further insight into the effectors of RANK^K240E^ signaling that mediate survival and proliferation, we analyzed the expression of the antiapoptotic molecule *Bcl-2* and the cell cycle regulator *Cyclin-D1*. Both factors are significantly higher expressed in RANK^K240E^-expressing B cells as compared with wt B cells from littermate control mice (Fig. 4 J). In addition, the expression of *Pten*, a negative regulator of the PI3K signaling pathway, was significantly reduced in RANK^K240E CD19Cre^ B cells as compared with wt B cells (Fig. 4 J), which is in line with the high levels of AKT phosphorylation observed in RANK^K240E^-expressing B cells (Fig. 4 H).

### RANK^K240E^ expression facilitates CLL development in aged mice

**Figure 5.**
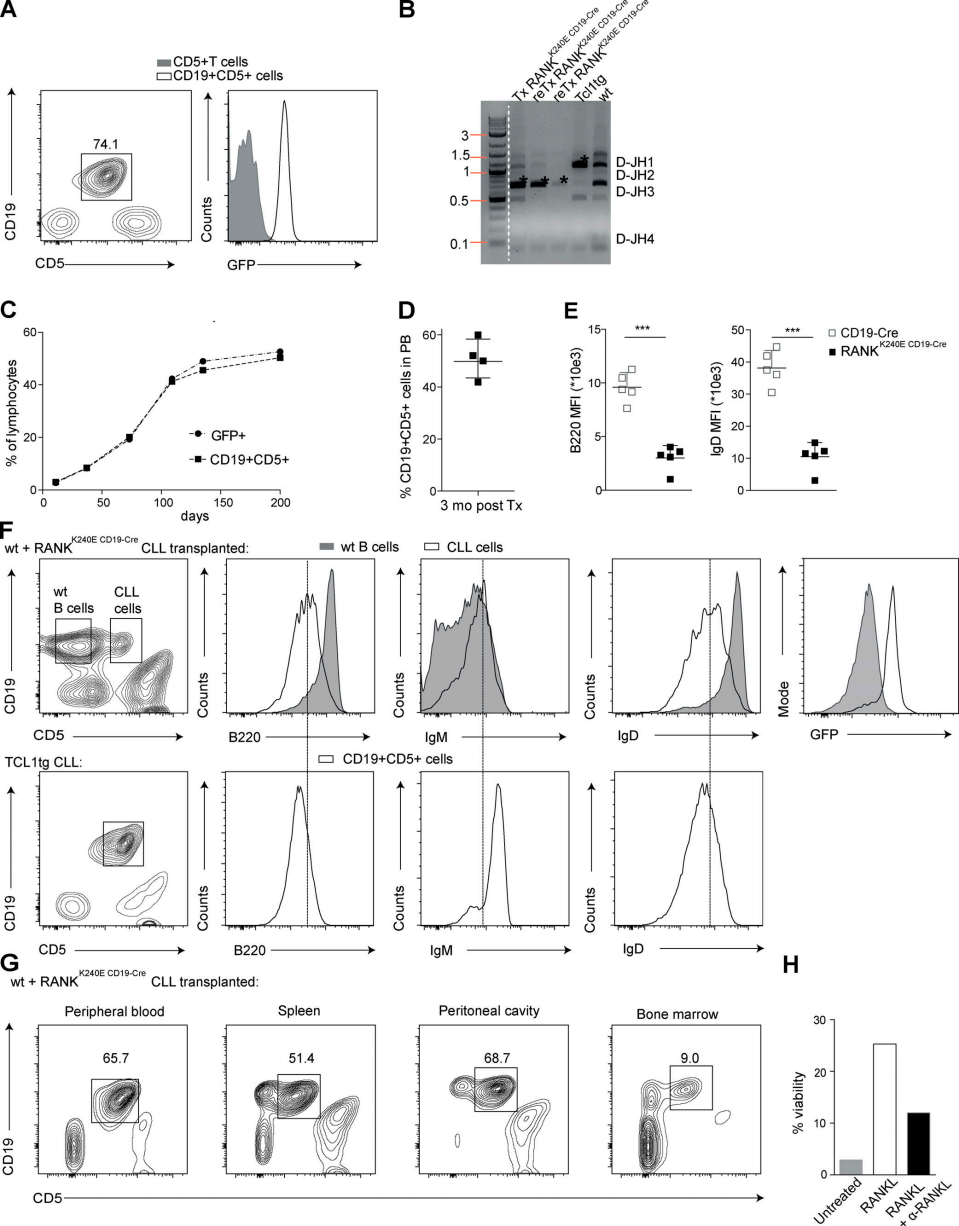
**Aged RANK^K240E^-expressing mice develop CLL.** **(A)** Flow cytometric analysis of splenocytes harvested from 12-mo-old RANK^K240E CD19-Cre^ mice revealed 74% CD19^+^CD5^+^ cells. GFP expression was confirmed. Representative for six >12-mo-old RANK^K240E CD19-Cre^ mice analyzed. **(B)** Ig clonality analysis of genomic DNA isolated from total peripheral blood of wt mice transplanted with splenocytes from an aged RANK^K240E CD19-Cre^ mouse, representative for three >12-mo-old RANK^K240E CD19-Cre^ mice analyzed. Tx, transplantation. reTx, retransplantation. **(C)** After transplantation of 2 × 10e7 splenocytes into wt recipients, the percentages of GFP^+^ and CD19^+^CD5^+^ cells were detected in the peripheral blood via regular blood draws. The engraftment of one of three different RANK^K240E CD19-Cre^ donor mice is shown. **(D)** Summary of percent CLL cells from three donors in the peripheral blood (PB) of four wt recipients, 3 mo after transplantation (Tx). **(E)** MFI values of B220 and IgD from three independent donors of RANK^K240E^-derived CLL cells in different wt recipients are shown. For statistical analysis, Student’s *t* test with paired analysis was performed; ***, P < 0.001. **(F)** CLL phenotypic analysis was performed in wt mice transplanted with cells from RANK^K240E CD19-Cre^ mice (representative for *n* = 5) and compared with that of classical TCL1-driven CLL (representative for *n* = 3). GFP expression was confirmed in the transplanted RANK^K240E^ transgenic CLL cells. **(G)** Organ distribution of CD19^+^CD5^+^ RANK^K240E^ transgenic cells in wt mice upon transplantation is shown; representative example for four recipient mice analyzed. **(H)** In vitro survival at day 10 in culture of CLL cells derived from an aged RANK^K240E CD19-Cre^ mouse in the presence and absence of RANKL and simultaneous RANKL blocking antibodies; representative for three independent experiments using two individual donor CLL from RANK^K240E CD19-Cre^ mice.

As indicated above, aberrant RANK signaling is potentially involved not only in autoimmunity but also in the pathogenesis of human B cell lymphomas. Therefore, we monitored aging RANK^K240E CD19-Cre^ mice for indications of B cell malignancy. Intriguingly, in RANK^K240E CD19-Cre^ animals that survived longer than 12 mo we detected dramatic accumulations of GFP^+^, CD19^+^, and CD5^+^ B1a cells (Fig. 5 A), which together constituted a homogeneous population. A PCR-based clonality analysis defining IgV_H_ D–J joinings of different sizes demonstrated that these populations originated from single dominant B1 cell clones (Fig. 5 B). In total, ∼20% of the RANK^K240E CD19-Cre^ mice lived longer than 12 mo, and we detected these expanded splenic CD19- and CD5-positive B cell subsets in all of these mice. To test whether the accumulated B1 cells were oncogenically transformed, we transplanted them into secondary nonirradiated, immunocompetent wt recipients and monitored the fate of these cells over time. In all cases, we observed an engraftment of the GFP^+^, CD19^+^, and CD5^+^ B1a cells in the secondary hosts and an accumulation of these cells over time (Fig. 5, C and D), demonstrating that they had acquired self-renewal capacity, which together with the clonal origin documents malignant transformation. Phenotypically, these transformed B1a cells resembled CLL cells, which characteristically coexpress CD19 and CD5 (Matutes et al., 1994) together with low surface expression of B220 and IgD (Fig. 5, E and F), and thus also phenocopied the malignant CLL cells of the classical TCL1-transgenic CLL mouse model (Fig. 5 F; Bichi et al., 2002). In addition, upon transplantation to secondary wt recipients, the transformed RANK^K240E^-expressing B1 cells accumulated in the blood, the spleen, and the peritoneal cavity and to a lesser extent in the bone marrow of wt recipients (Fig. 5 G) and thereby resemble the phenotypic behavior of adoptively transferred classical TCL1-transgenic CLLs (Hofbauer et al., 2011). To test whether the transformed RANK^K240E^-expressing CLL cells would still respond to exogenous RANKL, we then stimulated them with the ligand. Indeed, RANKL still provided a strong survival signal to these transformed CLL cells, which was blocked by a neutralizing anti-RANKL antibody (Fig. 5 H). Thus, chronically enforced RANK signaling within B cells facilitates CLL development, and the presence of exogenous RANKL is continuously required to maintain the survival of RANK^K240E^-expressing tumor cells.

### Murine and human CLL cell survival depends on RANK–RANKL signaling

**Figure 6.**
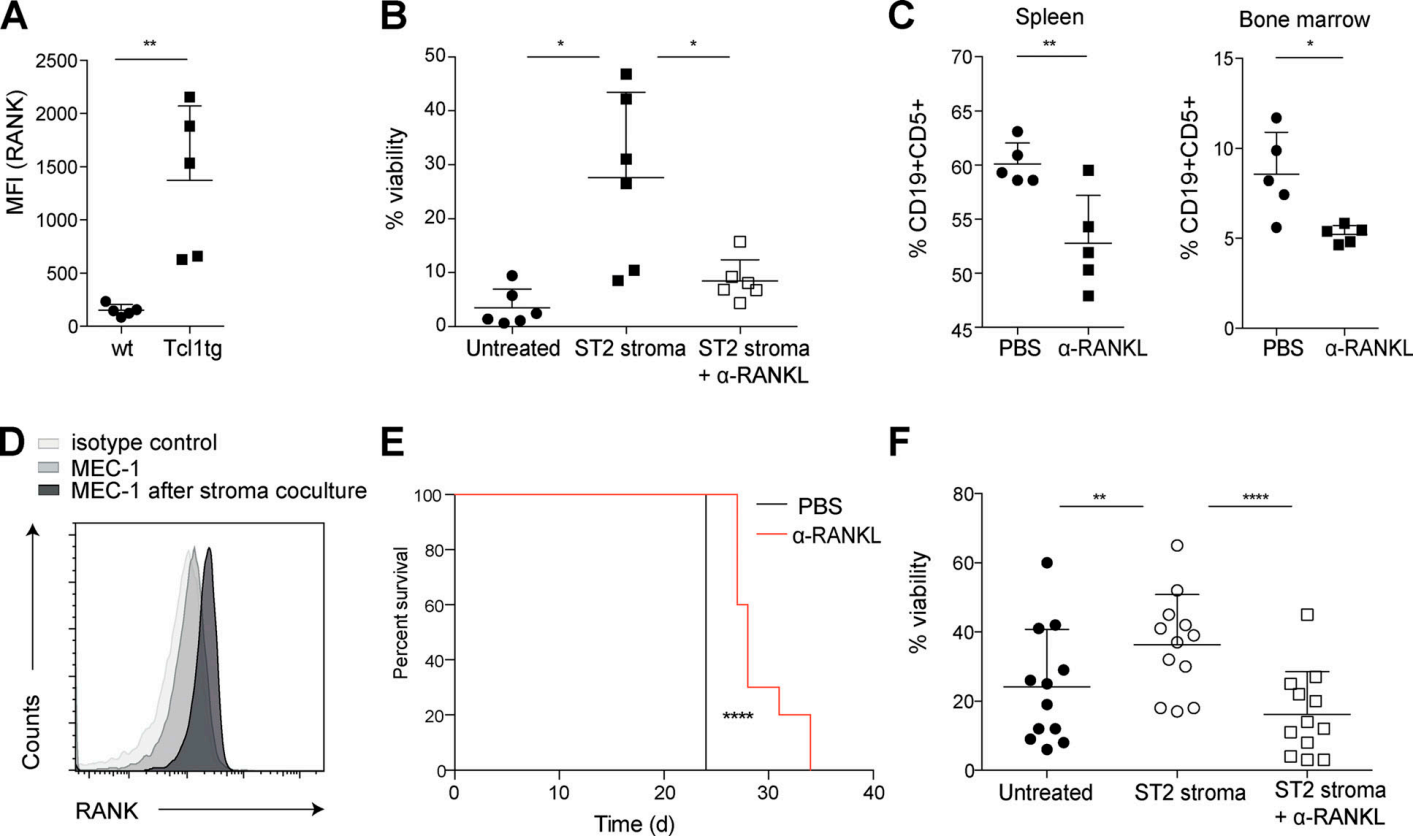
**RANK–RANKL is an important survival axis for CLL cells within their microenvironment. (A)** The MFI values of RANK expression on the surface of CD19^+^ WT B cells and CD19^+^CD5^+^ murine CLL cells derived from TCL1 transgenic aged mice (*n* = 5, measured in three independent experiments) are shown. **(B)** In vitro survival of murine CLL cells derived from five TCL1 transgenic donor mice in the presence and absence of the RANKL-expressing stromal cell line ST-2 with concomitant RANKL blocking antibody is shown, measured in two independent experiments. **(C)** Splenocytes and bone marrow cells were analyzed for CLL cell content after transplantation of TCL1-tg CLL into 10 wt recipients and administration of the RANKL blocking antibody twice a week (*n* = 5) or control (PBS, *n* = 5) for a total of 4 wk. **(D)** Surface RANK expression on MEC-1 cells was confirmed by flow cytometry with and without stromal cell contact for 24 h. Representative FACS analysis for three independent experiments is shown. **(E)** The CLL-derived MEC-1 cell line was transplanted into 10 NSG mice, and on day 8, RANKL treatment (*n* = 5) or control treatment (*n* = 5) was initiated twice a week. Kaplan-Meier analysis is shown, and for statistical analysis, log-rank (Mantel Cox) analysis was performed. ****, P < 0.0001. **(F)** Human patient–derived CLL cells derived from 12 individual donors were cocultured with the RANKL-expressing stromal cell line ST-2 in the presence and absence of RANKL blocking antibody. Viability was assessed in three independent experiments by DAPI exclusion via flow cytometry. For statistical analysis, Student’s *t* test was performed unless indicated otherwise. *, P < 0.05; **, P < 0.01; ****, P < 0.0001.

Because the crosstalk between malignant B cells and accessory cells in the microenvironment is of general importance for CLL tumor cell growth and disease progression (Burger, 2013), we next explored the role of RANKL–RANK signaling on a CLL background without the RANK^K240E^ transgene. To this end, we first studied RANK receptor expression on CLL cells from TCL1 transgenic mice (Bichi et al., 2002) and found that these malignant cells expressed high levels of RANK on their surface, with a mean 10-fold increase compared with peripheral wt CD19^+^ B cells from littermate control mice (Fig. 6 A). Subsequently, we cocultured CLL cells from six individual TCL1 transgenic mice with ST-2 stromal cells in the presence or absence of anti-RANKL antibodies. The ST-2 stroma strongly supported the viability of the CLL cells, and the addition of anti-RANKL significantly reduced this survival signal, indicating that supportive stroma effects are at least in part mediated via the RANKL–RANK axis (Fig. 6 B). Next, we treated a cohort of mice with TCL1 transgenic CLL with anti-RANKL antibodies. After 4 wk of treatment with the blocking RANKL antibody, we detected a significantly lower leukemia cell burden in the spleens and the bone marrow than that in the vehicle control (Fig. 6 C), which demonstrates that the RANKL–RANK axis contributes to the leukemia-supportive CLL microenvironmental crosstalk in vivo.

To explore the relevance of these findings in human CLL models, we then xenotransplanted the human MEC-1 CLL cell line into immunocompromised NOD/SCID/IL2rγ^−/−^ (NSG) mice and treated these animals with anti-RANKL antibodies. RANK expression on MEC-1 cells was confirmed and significantly up-regulated upon contact with ST-2 stromal cell (Fig. 6 D). Whereas all animals of the control group had to be euthanized 24 d after MEC-1 transplantation because of weight loss and hind limb paralysis due to infiltration of the MEC-1 cells into the spinal cord (Fig. 6 E), anti-RANKL treatment significantly extended the symptom-free survival for this aggressive, rapidly progressing human CLL xenograft model (Bertilaccio et al., 2013). Then, we investigated the supportive effects of the RANKL–RANK axis on primary CLL patient samples by incubating them with and without ST-2 stromal cells in the presence and absence of anti-RANKL. The CLL patient characteristics are shown in Table S1. Strikingly, while the ST-2 stromal cells supported the viability of the primary CLL cells, this supportive effect was largely abrogated by the anti-RANKL treatment (Fig. 6 F). Altogether, these results demonstrate that the RANKL–RANK signaling axis provides general microenvironmental survival support for both murine and human CLL cells in vitro and in vivo and that this leukemia-promoting signal can be disrupted using anti-RANKL antibodies.

## Discussion

In this study, we forced RANK^K240E^ expression in B cells to experimentally explore the consequences of aberrant RANK signaling in B cell immunopathology. The RANK^K240E^ receptor triggers potent cell survival and proliferation pathways in B lymphocytes in vitro and disrupts B cell tolerance in vivo*,* resulting in fully penetrant SLE-like autoimmunity with progression to B cell malignancy.

The pathological effect of the RANK^K240E^ receptor in B cells depends on the presence of exogenous RANKL, as these effects are inhibited by anti-RANKL treatment. In vivo*,* RANKL can be secreted by the bone marrow microenvironment and by activated T cells to stimulate RANK^K240E^ transgenic B cells. Our analyses show that wt B cells do not exhibit enhanced survival nor proliferation upon exposure to RANKL in culture, which is consistent with the fact that B cells that are conditionally deficient for RANK do not show a strong phenotype (Perlot and Penninger, 2012). However, stimulation of RANK^K240E^ by its ligand triggers strong activation of PI3K and JNK signaling in RANK^K240E^-expressing B cells. In line with this, inhibition of PI3K or JNK signaling prevented RANKL-mediated B cell activation and survival of RANK^K240E^-expressing B cells. The induction of PI3K signaling upon RANK ligation can occur directly via recruitment of TNF receptor–associated factors and activation of c-Src (Arron et al., 2001; Wong et al., 1999), while the repression of *Pten* that we observed may additionally contribute to sustaining PI3K signaling in RANK^K240E^-expressing B cells. Together, these data demonstrate that the PI3K pathway and the JNK pathway are dominant RANK^K240E^ effector cascades.

In vivo, chronic RANK^K240E^ signaling resulted in the expansion of B cells, particularly of the B1 subset, and in the loss of self-tolerance with an altered repertoire, which is demonstrated by the presence of serum autoantibodies and the autoimmune pathology in RANK^K240E CD19-Cre^ mice. Consistent with the hypothesis that these pathologies are also driven by aberrant PI3K signaling, similar phenotypes have previously been observed in other mouse models with enhanced PI3K activity (Anzelon et al., 2003; Suzuki et al., 2003). Downstream of PI3K activation, FOXO factors are inactivated in developing B cells (Fruman and Bismuth, 2009; Zhang et al., 2011). Therefore, RANK-mediated PI3K activation at B cell selection checkpoints could in theory prevent proper FOXO-mediated induction of the proapoptotic molecule Bim, which results in the survival of autoreactive B cells (Arron et al., 2001; Enders et al., 2003; Lau et al., 2020; Wong et al., 1999). This is likely one mechanism by which forced RANK^K240E^ signaling can disrupt B cell tolerance. In addition, RANK is also a strong activator of NF-κB (Anderson et al., 1997). We did not detect significant reduction of RANK^K204E^-mediated B cell survival using an NF-κB inhibitor nor substantial NF-κB activity in ex vivo–stimulated RANK^K204E^-expressing B cells (not shown), likely due to feedback regulation upon prolonged RANK^K204E^ activation (Ruland, 2011). Nevertheless, it is conceivable that pulsed RANK^K204E^-mediated NF-κB activity in vivo could enhance cell survival and proliferation in vivo and contribute to the loss of B cell tolerance in RANK^K240E^ transgenic mice (Grossmann et al., 2000; Guttridge et al., 1999; Hinz et al., 1999).

Our experimental strategy for B cell–specific RANK^K240E^ expression used the CD19-Cre transgene to induce RANK^K240E^ at the pre–B cell stage and throughout further development (Rickert et al., 1997). Normal developing B cells typically express only very low levels of RANK at this stage (https://www.immgen.org and our own mRNA analysis; data not shown). However, RANK can be induced on B lymphocytes, for example, by activated CD40L-expressing T cells (Yun et al., 1998). Therefore, it is possible that pathological RANK expression on individual human autoreactive B lymphocyte clones could mediate their survival upon RANKL binding and thereby disrupt central or peripheral immune tolerance and promote autoimmune disease. This hypothesis warrants further investigation but would be in line with the high levels of free RANKL in human SLE and RA patients (Carmona-Fernandes et al., 2011; Fonseca et al., 2005). Moreover, a recent study using murine RA models reported progressively increased RANKL levels in the diseased mice, which positively correlated with disease severity (Papadaki et al., 2019). While the genetic inactivation of RANKL dramatically attenuated arthritis, the overexpression of RANKL exacerbated RA in these animals (Papadaki et al., 2019). Since the RANKL–RANK axis is key for physiological bone remodeling and bone regeneration during inflammatory diseases, several effects of RANKL in RA mice are presumably due to the stimulation of osteoclasts. However, based on our results that demonstrate that pathological B cell–intrinsic RANK signaling can promote immunopathology, investigating additional effects of anti-RANKL treatment on B lymphocytes is important in RA models and in human autoreactive B cell responses. This is of particular interest, as the RANKL–RANK axis can be efficiently targeted in the clinic with the blocking anti-RANKL antibody denosumab, which is routinely used for the treatment of osteoporosis or osteolytic bone metastasis (Cummings et al., 2009; Henry et al., 2014) and is currently under investigation for RA treatment (Takeuchi et al., 2019).

In addition to autoimmunity, prolonged B cell–intrinsic RANK^K240E^ signaling promotes the development of B cell malignancy over time that resembles human B cell lymphoma and CLL and phenocopies classical CLL mouse models with characteristic surface marker expression and in vivo growth patterns (Bichi et al., 2002). This specific clonal CLL phenotype of our model exhibits self-renewal capacity, which is presumably facilitated by an initial RANK^K240E^-driven expression of survival and proliferation genes such as *Bcl-2* or *Cyclin-D1* and an expansion of tolerized B1 cells that represent the CLL progenitor population (Hayakawa et al., 2016). CLL cells frequently recognize autoantigens (Dühren-von Minden et al., 2012; Hamblin et al., 1999; Iacovelli et al., 2015), and their pathological BCR signals are indispensable for CLL development (Hayakawa et al., 2016). Under normal conditions, autoreactive BCR signaling triggers negative selection and deletion of the specific B cell clone (Hartley et al., 1991; Köhler et al., 2008). As pathological RANK^K240E^ signaling can overcome negative selection, these enforced survival signals are likely to support the growth of premalignant autoreactive B1 cells and thereby enable these cells to acquire additional genetic alterations leading to malignant transformation. RANK with its agonistic ligand RANKL induces the activation of B cell survival pathways in these cells. In addition, RANK^K240E^ was originally identified from human diffuse large B cell lymphoma, which typically originates from germinal center B cells (Küppers et al., 1999). It is thus conceivable that aberrant RANK signaling could also facilitate B cell malignancies at other developmental stages, and it is therefore important to explore the consequences of forced RANK expression selectively in B cells beyond the B1 stage in vivo*.*

In CLL, it is well established that the survival and expansion of tumor cells depends critically on close microenvironmental interactions with bystander cells (Burger, 2013). We have now identified the RANKL–RANK interaction as an important microenvironmental signal that promotes CLL development and CLL cell survival in the murine and human systems. High levels of RANK receptor are detected on the surface of human patient–derived CLL cells and on murine CLL cells (Schmiedel et al., 2013; Secchiero et al., 2006). Blocking anti-RANKL antibodies, which disrupt the RANKL–RANK interaction, thereby prevents prosurvival programs, inhibiting not only the survival of RANK^K240E^ transgenic CLL cells, but also TCL1 transgenic CLL cells, human CLL cell lines, and primary patient samples both in vitro and in vivo. Together, our findings not only demonstrate that aberrant RANK signaling contributes to the development of CLL in the early phases and upon experimental RANK^K240E^ transgene expression but also show that pathological RANKL–RANK signals mediate CLL tumor cell maintenance after malignant B cell transformation in a broader context. Together with the clinical availability of the blocking anti-RANKL antibody denosumab, our results warrant translational investigations targeting the RANKL–RANK axis for CLL treatment. Since microenvironmental up-regulation of prosurvival factors also contributes to the resistance of CLL cells to current drug therapies (Leverson and Cojocari, 2018; Munk Pedersen and Reed, 2004), the potential effects of anti-RANKL in counteracting chemoresistance or targeted therapy resistance should also be investigated.

In conclusion, our study provides mechanistic insights into the functions of aberrantly enforced RANK signaling in B cell–mediated autoimmunity and CLL pathogenesis. The codevelopment of these B cell pathologies in RANK^K240E^ transgenic mice is in line with the strong epidemiological link between human CLL and autoimmune manifestations (Barcellini et al., 2006; Duek et al., 2006; Vanura et al., 2008). These results indicate that aberrant RANKL–RANK signaling is a potential common mechanism of these pathologies. Our data further encourage exploring the potential of repurposing clinically available compounds that target the RANKL–RANK axis for the treatment of B cell–mediated autoimmunity and malignancies.

## Materials and methods

### Mice

Human RANK^K240E^ cDNA was cloned into the ubiquitously expressed ROSA26 vector, preceded by a loxP-flanked transcriptional and translational STOP cassette. ROSA26 was subsequently linearized and electroporated into 129Ola embryonic stem cells. The clones were verified by Southern blot analysis with a 5′ flanking ROSA26 probe and specific PCR, as previously described (Knies et al., 2015). Blastocyst injection of the clones and subsequent chimera breeding resulted in RANK^K240E^ ^stopFL^ mice, which were then crossed with CD19-Cre mice (Rickert et al., 1997). The bicistronic expression of RANK^K240E^ together with enhanced GFP preceded by an IRES sequence allowed monitoring of RANK^K240E^-expressing cells via fluorescence. Mice were backcrossed to C57/Bl6 mice for at least six generations, and littermates were used as controls in all experiments. The TCL1 transgenic mouse model (Bichi et al., 2002) was used to compare RANK^K240E^-derived leukemic cells and to study the effects of anti-RANKL treatment in an in vivo CLL transplantation setting. For murine transplantation experiments, recipient mice were ordered from Janvier Labs (C57/Bl6/N). For xenotransplant experiments, we used NSG mice (NOD.Cg-Prkd^cscid^ Il2rg^tm1WjI^/SzJ; purchased from Jackson Laboratories) as recipients for the human MEC-1 CLL-like cell line (purchased from Deutsche Sammlung von Mikroorganismen und Zellkulturen (DSMZ)). All animal work was conducted in accordance with German Federal Animal Protection Laws and approved by the Institutional Animal Care and Use Committee at the Technical University of Munich.

### Cell culture

The mature murine B cell lymphoma cell line Bal17 (RRID: CVCL_9474), freshly isolated primary B cells, isolated primary TCL1 transgenic CLL cells, and CD4^+^ T cells were cultured in RPMI-1640 medium supplemented with 10% FBS, 1% penicillin/streptomycin, 1% L-glutamine, and 0.1% 2-mercaptoethanol. ST-2 mouse bone marrow stromal cells and Phoenix-E and HEK293FT packaging cells were kept in DMEM supplemented as described above. Primary CLL patient–derived cells were cultured in RPMI-1640 Glutamax medium containing 10% FBS, 1% penicillin/streptomycin, 1% sodium butyrate, and 1% nonessential amino acids. The CLL-like MEC-1 cell line was cultured in IMDM supplemented with 10% FBS and 1% penicillin/streptomycin. All cells were cultured under standard cell culture conditions at 37°C in 5% CO_2_ and 95% humidity.

### Retroviral transduction

Human RANK^K240E^ and wt RANK were cloned into a pMSCV-IRES-GFP vector (Addgene plasmid # 27490; RRID: Addgene_27490) using standard techniques. Viral supernatants were produced by infecting Phoenix-E packaging cells as previously described (Knies et al., 2015). Bal17 cells were transduced with the viral supernatant by spin infection.

### Measurement of serum Ig and autoantibody levels

Detection of serum Ig was performed using the Mouse Immunoglobulin Panel, manufactured by Southern Biotech, using diluted sera as previously described (Knies et al., 2015). Detection of serum autoantibodies was performed according to the manufacturer’s instructions using Autoimmune ELISA Kits manufactured by Alpha Diagnostic International. Sera were diluted before use.

### Flow cytometry and FACS

Organs were processed into single-cell suspensions, treated with red blood cell lysis buffer, and resuspended and washed in FACS buffer (PBS, 3% FBS). After incubation with CD16/32 to block free F_c_ receptors, the cells were washed in FACS buffer again and incubated for 20 min at 4°C with fluorescently conjugated antibodies against surface molecules. All antibodies were diluted in FACS buffer. The cells were acquired using a FACS CantoII flow cytometer (BD), and the results were analyzed using FlowJo Software (Tree Star, Inc.). The following antibodies were used: B220 (RA3-6B2), CD80 (16-1QA1), CD86 (GL1), IgM (II/41), IgD (11-26c), CD19 (1D3), MHCII (M5/114.15.2), CD138 (281–2), CD4 (GK1.5), CD5 (53–7.3), CD8 (53–6.7), CD44 (IM7), CD62L (MEK-14), CD93 (AA4.1), RANK (CD265, 9A725; ThermoFisher Scientific), and RANKL (CD254, IK22/5; all from eBioscience unless stated otherwise). RANK^K240E^-expressing B cells were sorted for GFP^+^ cells for downstream applications. CD19-Cre B cells were stained with CD19 and sorted for CD19^+^ cells. Cells were sorted directly into filtered sterile FBS and washed with FACS buffer before use. Cell viability was quantified by flow cytometry with Annexin V and 7-AAD staining or DAPI (eBioscience), and cell proliferation was quantified by CellTrace Violet Cell Proliferation Kit (ThermoFisher Scientific) staining.

### Electron microscopy

Kidney biopsies were fixed for at least 2 h with 3% glutaraldehyde in Sörensen’s buffer, pH 7.4, washed three times in Sörensen’s buffer, post-fixed for 1 h in 1% osmium tetroxide, washed three times in Sörensen’s buffer, dehydrated by a graded series of ethanol, transferred into propylene oxide, and embedded in epon-araldite. Semithin and 70–80-nm ultrathin sections were cut with a Reichert Ultracut E ultramicrotome, counterstained with uranyl acetate and lead citrate, and examined with a JEOL 1400 transmission electron microscope equipped with a TVIPS F216 digital camera.

### Ig clonality and SHM assays

The clonality of the B cell repertoire in mice was determined using genomic DNA isolated from sorted B cells by PCR as described previously (Pennycook et al., 1993). SHM frequency was detected via a nested PCR method as previously described with a set of 5′ consensus primers for murine V_H_ or D segments of the heavy chain regions (Ehlich et al., 1994), followed by subcloning and sequencing of the amplified segments. The sequencing results were analyzed using the international ImMunoGeneTics information system (IMGT)/V-QUEST database.

### Indirect immunofluorescence HEp-2 assay

Single slides for indirect immunofluorescence (Euroimmun AG) were used according to the manufacturer’s instructions. In brief, the serum was diluted 1:100 in PBS and incubated on the slide for 30 min at room temperature. After 5 min of washing with PBS, 50 µl detection antibodies solution (1:400 Alexa Fluor 647 goat anti-mouse IgM [μ chain] and 1:400 Alexa Fluor 488 goat anti-mouse IgG [H+L] in PBS) was added and incubated at room temperature in the dark for 30 min. After 5 min of washing with PBS, the slides were covered with a cover glass and abundant PBS was removed. Images were recorded with a Leica DMRBE camera.

### Cell purification

For coculture experiments, CD4^+^ T cells were isolated from the spleens and lymph nodes of mice by negative magnetic activated cell sorting (MACS; Miltenyi Biotech). B cells from wt littermate mice or transgenic mice were sorted fluorescently. Primary human CLL cells were isolated from patient peripheral blood via Ficoll density gradient separation, and CLL cell content in peripheral blood mononuclear cells was determined by flow cytometric analysis of CD5 and CD19. Samples with CLL cell content >70% were used for experiments.

### Cell stimulation and inhibitor treatment

RANK^K240E^-expressing or wt B cells from littermates were stimulated with 100 ng/ml mouse recombinant RANKL (R&D Systems), 10 µg/ml neutralizing antibody (purified mouse α-RANKL; eBioscience), or 0.5 µM CpG (InvivoGen) for 1 to 48 h at 37°C. Following transduction, Bal17 cells were stimulated with 100 ng/ml mouse recombinant RANKL for 5 min to 48 h at 37°C. SP600125 was purchased from Sigma-Aldrich, and Cal-101 and PD0325901 (PD901) from Selleckchem, and BAY70-1182 was purchased from Caymanchem. The inhibitors were dissolved in DMSO and used at the indicated concentrations. Murine and human CLL cells were cultivated in coculture with murine bone marrow–derived stromal cells (ST-2) at a ratio of 10:1 and were treated with 10 µg/ml neutralizing antibody (purified mouse α-RANKL; eBioscience) for 24 to 72 h. To test RANKL dependency of transplanted RANK^K240E^-expressing CLL cells, peripheral blood cells derived from transplanted mice were stimulated with mouse recombinant RANKL (R&D Systems) and subsequently treated with neutralizing antibody (purified mouse α-RANKL; eBioscience) as described above and analyzed after 24 h and up to 10 d for survival via DAPI staining and flow cytometric analysis.

### Western blotting

Whole cell lysates were obtained using CHAPS lysis buffer, and protein concentrations were determined by the Bradford assay. 10 µg of the samples were used for Western blotting. The blots were probed with the following antibodies: phospho-JNK (no. 9251), phospho-AKT (no. 9275), phospho-ERK (no. 4370), β-actin (no. 3700), and phospho-PLC-γ2 (no. 3871; all obtained from Cell Signaling Technology).

### Quantitative real-time PCR

RNA was isolated from sorted RANK^K240E^-expressing and wt B cells by using RNeasy Mini Kit (QIAGEN) according to the manufacturer’s instructions. RNA concentration of the samples was determined by NanoDrop. RNA was reverse transcribed using SuperScript II (Invitrogen) according to the manufacturer’s instructions using a 20-µl reaction of 100 ng to 1 µg total RNA, 0.5 mM deoxynucleotide triphosphates, 250 ng random primers, 5 mM DTT, and 10 U/µl of SuperScriptTM II. The generated cDNA was used in duplicates or triplicates for RT-PCR reactions, with primers that span exon–exon boundaries to ensure cDNA-specific amplification. The qPCR Core Kit for SYBR Green I (Roche) was used to perform RT-PCR. Gene expression patterns were normalized to the housekeeping gene, *Actin*. The reaction was performed in a Light Cycler 480 II (Roche) and analyzed for quality using melting curves.

### Murine CLL model and anti-RANKL treatment

A total of 2 × 10^7^ diseased TCL1 transgenic splenocytes were intravenously injected into 10 female C57BL/6 mice (6–12 wk old; purchased from Janvier Labs). Tumor growth was monitored in the peripheral blood via flow cytometric analysis of CD19 and CD5. Upon detection of a CLL population, mice were randomized into a treatment and a control group, and the mice were treated intraperitoneally with 5 mg/kg anti-RANKL antibody (eBioscience) or PBS three times a week, respectively. After 4 wk, all mice were sacrificed, and secondary lymphoid organs were analyzed for CLL cell content.

### Patient samples

Primary CLL samples were obtained from the peripheral blood of patients at the National Center for Tumor Diseases, Heidelberg, Germany. Data for IGV_H_ status, ZAP70 expression, and time to first treatment were taken from the clinical records. The local ethics committee of the Faculty of Medicine, Technical University Munich, approved patient sampling, and all patients gave informed consent.

### Tumor xenograft model

MEC-1 cells (DSMZ Cat# ACC-497, RRID: CVCL_1870; 2 × 10^6^) were injected intravenously into NSG mice (8–12 wk old, female, *n* = 5 per group; purchased from Jackson Laboratory). On day 8 after transplantation, the mice were separated into two groups and received 5 mg/kg anti-RANKL or PBS twice per week (i.p.). The mice were sacrificed upon clear signs of disease according to the animal protocol guidelines.

### RANK^K240E^ CLL cell transplantation

Splenocytes (2 × 10^7^) of RANK^K240E CD19-Cre^ mice (>12 mo of age, containing >20% GFP^+^CD5^+^CD19^+^ cells) were transplanted into C57BL/6 recipients (purchased from Janvier Labs). Cell outgrowth was followed via flow cytometric analysis of CD19/CD5 and GFP in peripheral blood cells.

### Online supplemental material

Fig. S1 shows the strategy for the generation of RANK^K240E^ transgenic mice and confirmation of transgene and GFP reporter expression in B cells. Fig. S2 demonstrates comparable development of bone marrow, marginal, and follicular B cell subsets as well as splenic T cell subsets in RANK^K240E CD19-Cre^ mice and littermate controls. Fig. S3 shows RANKL expression detected by flow cytometry of immune and stromal cells, the effects of MAPK, NF-κB, PI3K, and JNK inhibitors on RANK^K240E^-mediated B cells survival and the expression of B cell activation markers CD86 and MHCII. Table S1 provides the CLL patients’ characteristics for the samples analyzed in this study, including their mutational status, sex, age at diagnosis, and the patients’ overall survival, as well as RANK (TNFRSF11A) and RANKL (TNFSF11A) mRNA expression levels.

## Supplementary Material

Table S1 depicts the CLL patients’ characteristics for the samples analyzed in this study, including their mutational status, sex, age at diagnosis, and the patients' overall survival, as well as RANK (TNFRSF11A) and RANKL (TNFSF11A) mRNA expression levels.
