# Supplementary material for: Pathological RANK signaling in B cells drives autoimmunity and chronic lymphocytic leukemia
Source: J Exp Med. 2020 Oct 14;218(2):e20200517. doi: 10.1084/jem.20200517 (PMC7868734; doi:10.1084/jem.20200517)
Supplement: Table S1 — depicts the CLL patients’ characteristics for the samples analyzed in this study, including their mutational status, sex, age at diagnosis, and the patients' overall survival, as well as RANK (TNFRSF11A) and RANKL (TNFSF11A) mRNA expression levels. [file JEM_20200517_TableS1.docx]

**Table S1. CLL patient characteristics**

| sample name | mutational status | sex | age at diagnosis | OS* (weeks) | TNFRSF11A mRNA expression | TNFSF11 mRNA expression |
| --- | --- | --- | --- | --- | --- | --- |
| CLL patient 1 | UM | f | 70 | 57 | 6.1 | 2.4 |
| CLL patient 2 | M | f | 46 | 70 | 16.2 | 4.9 |
| CLL patient 3 | M | m | 58 | 97 | 3.4 | 3.4 |
| CLL patient 4 | UM | m | 63 | 164 | 2.3 | 3.4 |
| CLL patient 5 | UM | m | 44 | 66 | 4.4 | 0.0 |
| CLL patient 6 | M | f | 57 | 110 | 20.2 | 4.0 |
| CLL patient 7 | M | f | 53 | 33 | 7.9 | 0.0 |
| CLL patient 8 | UM | m | 73 | 98 | 2.8 | 0.0 |
| CLL patient 9 | M | m | 58 | 94 | 7.1 | 0.0 |
| CLL patient 10 | M | m | 68 | 66 | 4.0 | 1.5 |
| CLL patient 11 | UM | m | 79 | 20 | 1.1 | 0.0 |
| CLL patient 12 | UM | f | 63 | 32 | 5.6 | 1.4 |

*OS: overall survival
